# Clarifying the relationship between alexithymia and subjective interoception

**DOI:** 10.1371/journal.pone.0261126

**Published:** 2021-12-13

**Authors:** Giulia Gaggero, Andrea Bizzego, Sara Dellantonio, Luigi Pastore, Mengyu Lim, Gianluca Esposito

**Affiliations:** 1 Department of Psychology and Cognitive Sciences, University of Trento, Rovereto, Italy; 2 Department of Education, Psychology, Communication, University of Bari, Bari, Italy; 3 Psychology Program, School of Social Sciences, Nanyang Technological University, Singapore, Singapore; 4 Lee Kong Chian School of Medicine, Nanyang Technological University, Singapore, Singapore; University College London, UNITED KINGDOM

## Abstract

The long-standing hypothesis that emotions rely on bodily states is back in the spotlight. This has led some researchers to suggest that alexithymia, a personality construct characterized by altered emotional awareness, reflects a general deficit in interoception. However, tests of this hypothesis have relied on heterogeneous assessment methods, leading to inconsistent results. To shed some light on this issue, we administered a battery of self-report questionnaires of interoception and alexithymia to three samples from Italy, the U.S., and Singapore (N = 814). Correlation and machine learning analyses showed that alexithymia was associated with deficits in both subjective interoceptive accuracy and attention. Alexithymics’ interoceptive deficits were primarily related to difficulty identifying and describing feelings. Interoception showed a weaker association with externally-oriented thinking as operationalized by the Toronto Alexithymia Scale (TAS-20) and no association with the affective dimension of alexithymia later introduced by the Bermond-Vorst Alexithymia Questionnaire (BVAQ). We discuss our results with reference to the theoretical and psychometric differences between these two measures of alexithymia and their shortcomings. Overall, our results support the view that interoceptive deficits are a core component of alexithymia, although the latter cannot be reduced to the former.

## Introduction

A long standing philosophical and psychological tradition has argued that somatic sensations play a role in the experience of emotions. Suffice it to mention the famous subtraction argument proposed by James and Lange [1, 2], who asked us to imagine experiencing a strong emotion without any of the feelings of the corresponding bodily symptoms. Their conclusion was that – without these bodily sensations – there was nothing left of the emotional experience. In the last decades, their intuitions about the essential role of bodily feelings in the identification of emotions have received renewed attention because they are consistent with theories of embodied cognition [3] and the work of neuroscientists like Damasio [4]. Proponents of these approaches argue that experiencing somatic changes is a prerequisite for emotional experience [5]. This view presupposes that, if a person has impaired interoception, that is an impaired ability to interpret and be aware of ongoing sensations inside their body, this will also limit their ability to experience emotions [6–8].

A subclinical condition called alexithymia gives us an opportunity to investigate this claim more closely. The term “alexithymia” (literally meaning “without words for emotions”, see [9]) was introduced by psychiatrists Nemiah and Sifneos [10, 11] to characterize a group of patients who manifested a general inability to verbalize their emotions due to a lack of emotional experience and their confusion between emotions and bodily states. The main features of the original construct of alexithymia included: (i) difficulty in identifying feelings and distinguishing between feelings and the bodily sensations associated with emotional arousal; (ii) difficulty in describing feelings to others; (iii) externally oriented thinking (a realistic style of thinking which avoids emotional expressions); and (iv) limited imaginal capacity [12]. The 20-item Toronto Alexithymia Scale (TAS-20; [13]), which is the currently the most widely used self-report instrument to assess alexithymia, was precisely designed to capture these features, which were respectively reflected in the first three factors: i) Difficulty Identifying Feelings (DIF); ii) Difficulty Defining Feelings (DDF); iii) Externally Oriented Thinking (EOT). These TAS-20 factors contribute to a total alexithymia score, coherent with the conceptualization of alexithymia as a condition that can be measured along a continuum.

This dimensional vision of alexithymia is not shared by all researchers. A more recent, but less widely used instrument to assess alexithymia, the Bermond-Vorst Alexithymia Questionnaire (BVAQ, [14, 15]), was developed on the basis of a categorical and modularist conceptualization of alexithymia. According to the researchers who developed BVAQ, there are two distinct brain modules that, when damaged, give rise to different types of alexithymia: one relates to the affective and the other to the cognitive aspect of emotions. These two alexithmia types are reflected in the independent factors measured by BVAQ: BVAQ-Cognitive and BVAQ-Affective [14, 16].

In practice, the BVAQ-Cognitive and TAS-20 can be considered highly comparable measures [14]: they capture the same facets of alexithymia and are highly correlated. By contrast, the BVAQ-Affective assesses limited imaginal capacity and reduced emotional arousal (operationalized by means of the “emotionalizing” factor). This last element represents the major novelty introduced by the BVAQ, since it implies that alexithymia does not only arise from a difficulty in achieving awareness of the experienced emotion but also from a difficulty in experiencing the emotion. It is still debated, if the latter relates to differences in physiological arousal [17], although this should be the primary reason for making a distinction between cognitive and affective types of alexithymia.

In spite of their differences, the TAS-20 and BVAQ presuppose the existence of a strong association between affective and somatic experience. A lack of interoceptive awareness is certainly reflected in the first factors of both scales (i.e., TAS-20 DIF; BVAQ Identifying), which aim to measure difficulty distinguishing between bodily and emotional feelings. Moreover, a deficit in interoceptive awareness could be hypothesized to underly the characteristics that define the “emotionalizing” factor of BVAQ, if this factor reveals differences in physiological arousal. It is, therefore, not surprising that, in the last ten years, researchers have started to more deeply explore the extent to which interoception contributes to the etiology of alexithymia.

The most radical proponents of this stream of research have formulated the “interoceptive hypothesis” of alexithymia. They argue that alexithymia is a general deficit in interoceptive ability [18], rather than a multifaceted construct that, among its core features, involves a general confusion between bodily and affective states. Their research has mainly focused on the Autism Spectrum Disorder population, providing evidence that alexithymia, which frequently co-occurs with autism, is responsible for both the interoceptive and emotional deficits associated with this neurodevelopmental disorder [19–24]. They have also explored the association between alexithymia and interoception in other clinical populations as well as in the general population [18, 25, 26]. Considering their findings alongside those of other research groups, they conclude that “alexithymia can be considered a proxy of atypical interoception” [27], characterized by either atypically low or high sensitivity. This suggests that alexithymia cannot occur without a deficit in interoceptive ability such that emotional deficits found across a wide range of clinical populations could be reduced to a deficit in interoception, defined as the “p-factor underlying susceptibility for psychopathology” [27].

Although the association between alexithymia and interoception is sound, this strong version of the “interoceptive hypothesis” faces numerous theoretical and methodological issues. On the one hand, we can question whether interoceptive ability is related to all features of alexithymia – independently of whether we embrace the dimensional view subtended by the TAS-20 or argue that there are different types of this condition as suggested by the BVAQ. This point has not been adequately explored, since the majority of studies that examine the relationship between alexithymia and interoception have relied on the TAS-20 to assess alexithymia, and have only rarely adopted other measures such as the BVAQ. On the other hand, we need to resolve some outstanding issues concerning the operationalization of interoception, as detailed in the next sections.

Khalsa and colleagues [28] have noted that interoception is a too broad concept. Interoceptive processing occurs across a variety of physiological systems (e.g., cardiovascular, pulmonary, gastrointestinal, genitourinary), many of which function outside the realm of conscious awareness. A number of self-report measures and behavioral tasks have been developed in an attempt to measure our ability to self-monitor aspects of aware interoception. Within this framework, interoception is described as implying three independent but interacting components: (1) *objective interoceptive accuracy* (IAcc), (2) *interoceptive sensibility* (IS), and (3) *interoceptive awareness* (IA) [29]. IAcc (1) refers to behavioral measurement of performance in interoceptive tasks such as heartbeat detection [30]. IS (2) refers to the self-evaluated assessment of subjective interoception, that is the extent to which one believes one can accurately perceive one’s own bodily states. IA (3) represents metacognitive awareness of interoceptive accuracy, that is the extent to which one’s interoceptive sensibility reflects one’s interoceptive accuracy. Subsequent models have adopted a more complex taxonomy, further distinguishing between these three components. For instance, Murphy and colleagues argued that both subjective and objective interoceptive accuracy and interoceptive attention should be assessed by different measures [31, 32].

Distinguishing between the different components of interoception is also useful for investigating the relationship between interoception and alexithymia since it helps to make sense of the apparently contradictory findings from different empirical studies. This was clearly revealed by a recent meta-analysis by Trevisan and colleagues [33] that considers different measures of interoception separately and investigates how (1) Objective Interoceptive Accuracy (Objective IAcc) (2) Subjective Interoceptive Accuracy (Subjective IAcc) and (3) Subjective Interoceptive Attention (Subjective IAtt) relate to alexithymia.

First of all, this meta-analysis shows that the association between Objective IAcc and alexithymia is non-significant. The explanation the authors give for this result is that the majority of the examined studies employed the heart-beat counting task [30, 34]: although this is the most widely employed measure of Objective IAcc, heart-beat counting can be influenced by numerous confounding factors; moreover, since it is domain-specific, it cannot capture the interoceptive ability as a whole [25, 35].

Secondly, Trevisan’s meta-analysis [33] examines the correlation between alexithymia and self-report measures of interoception, which fall either in the dimension of Subjective IAcc or in that of Subjective IAtt. This analysis confirms that alexithymia and Subjective IAcc – as measured by questionnaires that assess perceived accuracy in detecting different internal signals – are moderately correlated (r(22) = .437, p<0.001). By contrast, the relationship between alexithymia and questionnaires that measure a self-perceived dispositional tendency to focus on interoceptive signals (Subjective IAtt) is not significant. However, they also note that measures of Subjective IAtt – i.e., the Multidimensional Assessment of Interoceptive Awareness (MAIA; [36, 37]), the Body Perception Questionnaire (BPQ; [38]) – show significant correlations with alexithymia, but one is negative while the other is positive, possibly explaining the overall non-significant result. In a further commentary, Trevisan, Mehling and Portland [39] elaborate on this point, suggesting that the two measures respectively operationalize adaptive or maladaptive forms of interoceptive attention and conclude that this is a source of confusion when it comes to assessing the relationship between overall interoception and alexithymia. This thesis remains speculative and, as the authors suggest, "there is an urgent need for further convergent validity studies on commonly used subjective interoception measures” and, in our view, also on a more shared and transparent way to rely on the alexithymia measures.

### The present study

This study aims to explore the extent of the association between subjective interoceptive ability and alexithymia. It places special focus on the still unresolved issues mentioned above.

Specifically, our purpose is to clarify:

(1a) Whether subjective interoceptive ability is associated only with alexithymia as it is operationalized by the TAS-20 and BVAQ-Cognitive or whether it also associated with the so-called Affective-type of alexithymia (operationalized by the BVAQ-Affective).
  (1b) Whether all or only some facets of alexithymia included in both the TAS-20 and BVAQ-Cognitive and Affective (e.g., DIF, DDF, Emotionalizing, etc.) are associated with subjective interoceptive ability.
Which dimensions of subjective interoception provide better estimates of alexithymia levels; i.e., is alexithymia best predicted by the Subjective IAcc (perceived accuracy in detecting your own internal bodily sensations) or by the Subjective IAtt (dispositional attention to internal bodily sensations).

Moreover, to assess whether our results are broadly replicable, we collected data from three large samples, which differed in terms of cultural and socio-demographic variables, and analysed them using two different statistical methods. Correlation analyses were used to compare our results to previous findings based on this same statistical method, while Machine Learning (ML) algorithms were applied to provide further data-driven confirmation of the results obtained from traditional inferential statistics (e.g., see [40, 41]).

## Materials and methods

Data were collected online from three separate sources: an Italian opportunity sample, a Singaporean University sample, and a U.S. sample recruited from Amazon’s Mechanical Turk (MTurk) platform. Data collections were approved by the IRBs of the University of Trento (Protocol n. 2020-029) and the Nanyang University of Singapore (PSY-IRB 2019-030; IRB 2020-10-016). Prior to data analysis, this study was preregistered (https://osf.io/5x9sg/) in the Open Science Framework (OSF).

### Participants

#### Italian sample

Participants were students recruited from the Universities of Trento, Perugia, and Bari, or via social media platforms. No monetary compensation was provided but participants from the University of Trento could request university credits for time spent completing the tasks. Inclusion criteria comprised age (>18) and language (Italian native speakers). The final sample entered in the analyses included 325 subjects (221 female, 102 male, 2 other; M_age_ = 23.49, SD_age_ = 4.57, age-range = 18–53; 80% university students, 20% workers/other).

#### U.S. sample

Participants recruited through Amazon’s Mechanical Turk were English‐speaking USA residents aged over 18. An additional inclusion criterion was at least a bachelor level education, although this last criterion was only assessed by self-report. Respondents received $6 for completing the survey. This data collection was approved by NTU’s 2020-10-016 IRB. The final sample entered in the analyses comprised 250 subjects (120 female, 130 male; M_age_ = 29.5, SD_age_ = 5.05, age-range = 22–58; 68% White Caucasian; 12% African American; 12% Asian / Pacific Islander; 6% Hispanic American; 2% Multiple Ethnicity/Other).

#### Singaporean sample

Participants were recruited from undergraduate students at NTU and received university credits for their participation. The final sample entered in the analyses comprised 239 subjects (150 female, 89 male; M_age_ = 21.8, SD_age_ = 2.08, age-range = 18–28; 82% Chinese, 7% Indian, 4% Malay, 2% Eurasian, 4% Other).

### Measures

#### Self-report measures of alexithymia

*Toronto-Alexithymia Scale (TAS-20; [13]).* The TAS-20 comprises 20 items rated on a 5-point Likert scale ranging from 1 (strongly disagree) to 5 (strongly agree). The items load on three factors: (i) Difficulty Identifying Feelings (DIF), e.g., “I am often confused about what emotion I am feeling”, (ii) Difficulty Describing Feelings (DDF), e.g., “It is difficult for me to find the right words for my feelings” and (iii) Externally Oriented Thinking (EOT), e.g., “I prefer talking to people about their daily activities rather than their feelings”. Total scores range from 20 to 100 points, where higher scores are indicative of higher levels of alexithymia.

*Bermond-Vorst Alexithymia Questionnaire (BVAQ; [14]).* The BVAQ consists of 40 items, rated on a 5-point Likert scale, where higher scores indicate higher levels of alexithymia. The items load on five subscales: (i) Identifying (i.e., the degree to which one is able to define one’s arousal states) (ii) Verbalizing (i.e., the degree to which one is able or inclined to describe or communicate about one’s emotional reactions.), (iii) Analyzing (i.e., the degree to which one seeks out explanations of one’s own emotional reactions), (iv) Fantasizing (i.e., the degree to which someone is inclined to fantasize, imagine, or day-dream), (v) Emotionalizing (i.e., the degree to which someone is emotionally aroused by emotion inducing events). In addition, Vorst and Bermond [14] revealed two higher-order orthogonal factors: a Cognitive component (comprising “Identifying”, “Verbalizing”, and “Analyzing” subscales), and an Affective component (comprising “Fantasizing” and “Emotionalizing”). Bermond et al. [16] suggested that the two dimensions (Cognitive and Affective) are partially independent and, therefore, decided to identify different types of alexithymia: Type I (i.e., severe reductions in both the Cognitive and Affective dimensions [COG -; AFF -]), Type II alexithymia (i.e., severe reductions only in the Cognitive dimension [COG -; AFF +]), Type III alexithymia (i.e., severe reductions only in the Affective dimension [COG +; AFF -].

#### Self-report measures of interoception

*Interoceptive Confusion Questionnaire (ICQ; [18])*. The ICQ assesses the degree to which individuals experience difficulty interpreting their own non-affective interoceptive states, such as hunger, temperature and arousal (e.g., “I frequently forget to eat”; “When I adjust the heat of a room or car, others find it uncomfortable”). It consists of 20 items rated on a 5-point Likert scale, ranging from 1 (Does not describe me) to 5 (Describes me very well). The overall interoceptive confusion index ranges from 20 to 100, where higher scores are representative of more difficulty with interoception. The questionnaire has no internal subdivisions.

*Interoceptive Accuracy Scale (IAS; [42])*. The IAS consists of 21 items rated on a 5-point Likert scale, ranging from 1 (strongly disagree) to 5 (strongly agree). Each item assesses self-reported accuracy (“I can always accurately perceive”) in the perception of a specific body signal (e.g., heartbeat, breath, anger, etc.). Total scores range from 21 to 105, where higher scores indicate greater self-reported interoceptive accuracy. The questionnaire has no internal subdivisions, although the initial factor analysis performed by Murphy et al. [32] suggested a two-factor solution that requires further scrutiny. The IAS was designed to overcome the low psychometric properties of the ICQ, probably due to the fact that the specificity of ICQ items (e.g., “I frequently forget to eat”) does not capture the broader difficulties an individual may have perceiving internal sensations (e.g., a person may experience difficulties perceiving hunger, but this may manifest in overeating rather than forgetting to eat).

*Multidimensional Assessment of Interoceptive Awareness - Version 2 (MAIA-2; [37])*. MAIA-2 consists of 37 items grouped in eight subscales: 1) Noticing (i.e., awareness of body sensations); 2) Not-Distracting (i.e., a tendency not to ignore or distract oneself from sensations of pain or discomfort; 3) Not-Worrying (i.e., a tendency not to worry about sensations of discomfort); 4) Attention Regulation (i.e., an ability to sustain and control attention to body sensations); 5) Emotional Awareness (i.e., awareness of the connection between body sensations and emotional states); 6) Self-Regulation (i.e., an ability to regulate distress by paying attention to body sensations); 7) Body Listening (i.e., listening to the body for insight); 8) Trusting (i.e., trusting one’s body sensations). Responders assess how often each statement applies to their experience in daily life on a scale ranging from 0 (never) to 5 (always). Scores for each subscale are derived by computing the individual mean of each subject on the items composing the specific subscale. A total index of interoceptive awareness is derived by summing the individual scores at each of the 8 subscales.

*Body Perception Questionnaire (BPQ; [38]).* The BPQ is a self-report instrument used to assess body perception and interoceptive awareness. For this study, we used the BPQ Short Form, consisting of 46 items. Each item is rated on a five-point Likert scale ranging from 1 (never) to 5 (always). Items are divided into two main domains: Body Awareness and Autonomic Reactivity. Items in the first domain measure subjective perceived sensitivity to internal bodily functions. Values at the high end of the scale reflect perceived hypersensitivity and values at the low scale reflect hyposensitivity. Items in the second domain measure the respondent’s perception of the reactivity of his/her autonomic system.

There were two main reasons why we selected these four interoceptive measures from among many others available in the literature. The first is that both the IAS and ICQ were developed by the research group that formulated the *“interoceptive hypothesis”* of alexithymia. Therefore, these measures represent crucial evidence for evaluating this hypothesis. This group specifically suggested that IAS and ICQ are measures of Subjective IAcc and should not be confused with measures of Subjective IAtt [32]. So, we also choose to examine the two subscales that are generally considered to measure Subjective IAtt, mainly MAIA and BPQ. In the subsequent Discussion, we justify our concerns regarding BPQ-Reactivity as a measure of interoception. However, no studies to date have analysed the two subscales of BPQ separately. By administering all BPQ items and conducting a separate analysis of the two subscales, we were able to clarify this point.

All the described measures were administered to each of the three samples. The English version was given to the Singaporean and the U.S. samples, while Italian translations were provided for the Italian sample. Validated translations in the Italian language were available for TAS-20 [43], BVAQ [15], MAIA-2 [44] and BPQ [45]. An Italian translation of IAS and ICQ was done by the authors of the present article (G.G; S.D) and back translation was provided by an English native-speaker.

### Data analysis plan

Data analysis was conducted using RStudio (version 1.3.1093) and Python (version 3.8) scikit-learn library (version 0.24, [46]). Preliminary analyses included calculation of Cronbach’s alpha and descriptive analyses of the socio-demographic and psychological data. The association between alexithymia and interoception was investigated by adopting two methodological frameworks: (i) correlation analyses between measures of interoception and alexithymia (ii) ML modeling to estimate alexithymia levels starting from measures of interoception.

#### Machine learning analysis

In this study, ML models were used to estimate the three alexithymia scores (namely: TAS-20 total scores, BVAQ-Cognitive and BVAQ-Affective) based on interoceptive scores. Specifically, Support Vector Machine (SVM) regressors with a linear kernel were used. SVMs are supervised learning methods used for either classification or regression. SVMs offer advantages over other ML methods because they are (i) effective in high dimensional spaces and (ii) not computationally demanding since they use a subset of training points in the decision function (called support vectors).

The same protocol was used to calibrate the three ML models, with each model estimating one of the three target variables. Firstly, the Italian dataset was randomly split into ‘train’ (75% of the subjects) and ‘test’ partitions (25% of the subjects). The ‘train’ partition from the Italian dataset was used to calibrate the models. The calibrated model was then evaluated on the remaining subsets: the Italian Test partition, the U.S. partition, and the Singaporean partition.

The comparison of Mean Absolute Error (MAE) in the three ML models led to an unbiased data-driven quantification of how interoceptive scales and subscales were associated with the different measures of alexithymia. By considering the replicability of the results across the three samples (Italian, U.S., and Singaporean), we could also assess how the findings generalized across different demographic groups. Finally, we used the rankings of the interoceptive scores computed by the ML models to obtain an indication of how different dimensions of Subjective IAcc (i.e., self-reported interoceptive accuracy and attention) were linked to the alexithymia scores.

A detailed description of all analytical steps including data pre-processing and further details on the ML models are available in S1 File.

## Results

Table 1 shows Cronbach’s alpha values for each measure and its subscales. In all three samples TAS-20 EOT and MAIA Noticing gave Cronbach’s alpha values under the standard acceptance levels (α <0.7). ICQ, which in the original paper ([18]) showed low Cronbach’s alpha (α = 0.53), was above or close to standard acceptance levels (α_italy_ <0.68; α_U.S._ <0.77; α_Singapore_ <0.66). All the other measures revealed alpha values above the acceptance level.

**Table 1:** Cronbach’s alpha values computed on each variable.

Scales/Subscales	Italy (n = 325)	U.S. (n = 250)	Singapore (n = 239)
TAS-20	0.86	0.89	0.83
TAS-20 DIF	0.85	0.9	0.84
TAS-20 DDF	0.83	0.83	0.8
TAS-20 EOT	**0.58**	**0.58**	**0.51**
BVAQ Identifying	0.85	0.84	0.81
BVAQ Verbalizing	0.88	0.89	0.87
BVAQ Analyzing	0.77	0.79	0.70
BVAQ Fantasizing	0.80	0.84	0.82
BVAQ Emotionalizing	**0.68**	0.72	0.77
BVAQ-Cognitive	0.89	0.91	0.87
BVAQ-Affective	0.73	0.81	0.79
IAS	0.85	0.9	0.88
ICQ	**0.68**	0.77	**0.66**
MAIA	0.88	0.91	0.85
MAIA Noticing	**0.56**	**0.68**	**0.58**
MAIA Not-Distracting	0.70	0.86	0.82
MAIA Not-Worrying	0.81	0.78	0.74
MAIA Attention Regulation	0.84	0.87	0.80
MAIA Emotional Awareness	0.85	0.84	0.75
MAIA Self-Regulation	0.81	0.86	0.80
MAIA Body Listening	0.83	0.89	0.82
MAIA Body Trusting	0.88	0.89	0.85
BPQ	0.92	0.95	0.91
BPQ-Awareness	0.92	0.95	0.92
BPQ-Reactivity	0.86	0.95	0.89

In bold are displayed values under acceptance level (<0.70).

Descriptive statistics of the socio-demographic and psychological data performed separately for the Italian, U.S. and Singaporean are summarized in Table 2. For information purposes, we reported comparative analyses of variance of the samples’ sociodemographic and psychological measures, although the main aim of this study was not to compare the samples with each other but to verify whether our results were consistent across different socio-demographic profiles. Results from one-way permutation ANOVAs were significant for all psychological variables, with the exception of IAS. However, effect sizes were small for all psychological measures (ηp^2^ < = 0.04). Post-hoc analysis revealed higher differences between the Italian sample and the other two samples, while they failed to find a significant difference between the U.S. and the Singaporean samples in any psychological measure, with the exception of MAIA total scores (p<0.01). Results from one-way permutation ANOVAs were also significant for age, which was the only variable to show a large effect size (ηp^2^ = 0.37). Age was also found to significantly differ in all post-hoc comparisons (p = 0.001). Results from the chi-square test also revealed significant differences in the proportion of male and females between the three datasets ((p<0.001).

**Table 2:** Mean (standard deviation in parentheses) values of age and psychological measures for the Italian, U.S., and Singaporean sample.

	*M (SD)*			F	Perm. P	*ηp* ^*2*^	Italy–U.S.	U.S.–Singapore	Italy–Singapore
	Italy (n = 325)	U.S. (n = 250)	Singapore (n = 239)						
**age**	23.49 (4.57)	29.52 (5.05)	21.81 (2.08)	236.02	0	0.37	0.001	0.001	0.001
**TAS-20**	48.34 (12.12)	51.38 (12.46)	51.72 (9.89)	7.46	0.001	0.02	0.011	1	0.003
**BVAQ-Cognitive**	57.93 (14.15)	60.78 (14.98)	61.92 (12.2)	6.31	0.002	0.01	0.059	1	0.002
**BVAQ-Affective**	35.63 (7.79)	37.81 (9.48)	38.41 (8.44)	8.51	0	0.02	0.009	1	0.001
**IAS**	80.80 (9.68)	80.90 (11.39)	82.57 (9.38)	2.46	0.083	0.01	1	0.254	0.077
**ICQ**	46.45 (8.74)	47.78 (10.37)	49.84 (8.56)	9.30	0	0.02	0.303	0.055	0.001
**BPQ-Awareness**	59.31 (6.62)	57.04 (8.84)	58.18 (6.56)	6.79	0.001	0.02	0.002	0.337	0.141
**BPQ-Reactivity**	55.21 (6.13)	52.82 (9.01)	51.68 (7.63)	16.28	0	0.04	0.002	0.411	0.001
**MAIA**	21.88 (4.92)	23.22 (5.17)	21.83 (3.87)	7.23	0.001	0.02	0.005	0.003	1

F statistics, P value, and eta squared (ηp2) of one-way permutation ANOVAs performed for each variable. The last three columns show reported p-values for post-hoc permutation t-tests with Bonferroni correction. P values are rounded to 3 decimal places.

### Correlation analyses

Zero-order Spearman’s correlations for each sample are displayed in Table 3. Correlations among all subscales are reported in the S1 File (S1-S3 Tables). In accordance with Cicchetti et al. [47], the magnitude of the correlation coefficients was evaluated as small (0.10–0.29), medium (0.30–0.49), large (0.50–0.69), or very large (≥ 0.70).

**Table 3:** Correlation matrices p < .05, ** p < .01, *** p < .001.

**Dataset Italy**							
**Variable**	1	2	3	4	5	6	7
TAS-20							
BVAQ-Cognitive	0.84***						
BVAQ-Affective	0.07	0.14					
IAS	-0.31***	-0.34***	0				
ICQ	0.46***	0.50***	-0.06	-0.53***			
BPQ-Awareness	-0.09	-0.17*	-0.17*	0.26***	-0.08		
BPQ-Reactivity	0.28***	0.26***	-0.17*	-0.31***	0.39***	0.29***	
MAIA	-0.40***	-0.44***	0.03	0.35***	-0.36***	0.23***	-0.03
**Dataset U.S.**							
**Variable**	1	2	3	4	5	6	7
TAS-20							
BVAQ-Cognitive	0.85***						
BVAQ-Affective	0.14	0.20*					
IAS	-0.34***	-0.32***	0.02				
ICQ	0.69***	0.62***	0.05	-0.47***			
BPQ-Awareness	0.07	0.02	-0.09	0.13	0.06		
BPQ-Reactivity	0.46***	0.37***	-0.07	-0.28***	0.59***	0.31***	
MAIA	-0.42***	-0.46***	0.18	0.40***	-0.34***	0.20*	-0.15
**Dataset Singapore**							
**Variable**	1	2	3	4	5	6	7
TAS-20							
BVAQ-Cognitive	0.68***						
BVAQ-Affective	-0.11	0.06					
IAS	-0.22**	-0.24**	0.02				
ICQ	0.46***	0.46***	-0.19*	-0.46***			
BPQ-Awareness	-0.02	-0.09	-0.15	0.20*	-0.17		
BPQ-Reactivity	0.38***	0.33***	-0.21*	-0.21*	0.41***	0.15	
MAIA	-0.36***	-0.43***	0.11	0.38***	-0.44***	0.18	-0.16

#### Correlations between measures of alexithymia

In all three samples there was a large correlation between the TAS-20 and BVAQ-Cognitive. The correlation between the BVAQ-Cognitive and BVAQ-Affective was not significant in either the Italian or the Singapore dataset and small in the U.S. sample. Similarly, there was no significant correlation between the TAS-20 and BVAQ-Affective in any dataset.

#### Correlations between measures of interoception

Regarding the relationship between different self-report interoceptive measures, a large (negative) correlation was found between IAS and ICQ across the three samples. Medium correlations were found between MAIA total scores and IAS or between MAIA total scores and ICQ in all samples.

The two dimensions of the BPQ showed a significant if moderate correlation in two out of the three datasets. However, correlating the two dimensions of the BPQ with the other scales of interoception, we obtained a different pattern of results. Indeed, BPQ-Reactivity always showed a significant negative correlation of medium magnitude with IAS and a significant positive correlation of medium-large magnitude with ICQ, but was not significantly correlated with MAIA total scores. By contrast, BPQ-Awareness showed an unstable pattern of results across the three samples and the direction of correlation with IAS, ICQ and MAIA was opposite to that between the same interoceptive measures and BPQ-Reactivity. For instance, in two out of three datasets, BPQ-Awareness showed a significant positive correlation of small magnitude with IAS and MAIA, while correlation with ICQ was never significant.

Overall, the correlations between IAS, BPQ-Awareness, and MAIA total scores all went in the same direction but in the opposite direction to ICQ and BPQ-Reactivity.

#### Correlations between measures of Interoception and Alexithymia

The relationship between measures of interoception and alexithymia as measured by the TAS-20 and BVAQ-Cognitive revealed a similar pattern of results. ICQ was the measure of interoception that showed the highest positive correlation with both the TAS-20 or BVAQ-Cognitive across the three samples. By contrast, BPQ-Awareness was the only measure of interoception that failed to show any overall significant correlation with either the TAS-20 or BVAQ-Cognitive. As for the correlation between the other measures of interoception and the TAS-20 or BVAQ-Cognitive, a consistent pattern of results emerged across the three samples, with a negative correlation of medium magnitude for both MAIA total scores and IAS and a positive correlation of medium magnitude for BPQ-Reactivity. Among the eight MAIA subscales (see S1-S3 Tables in S1 File), Body Trusting showed the highest negative correlation with both the TAS-20 and BVAQ-Cognitive across all three samples. In the U.S. and Italian datasets, all the MAIA subscales showed a significant negative correlation with the TAS-20, while in the Singaporean sample three MAIA subscales (Body Listening, Emotional Awareness and Noticing) did not significantly correlate with the TAS-20. Correlation analyses between each of the eight MAIA subscales and the BVAQ-Cognitive showed a consistent pattern of results across all three samples: all MAIA subscales negatively correlated with the BVAQ-Cognitive with the exception of Not-Worrying.

Moving to the analysis of correlations between BVAQ-Affective and measures of interoception, it is worth noticing that correlations were not-significant or, when they reached significance levels (*), were of small magnitude and went in the opposite direction to correlations between these same interoceptive scales and the TAS-20 or BVAQ-Cognitive. The only exception was BPQ-Awareness in the Italian sample, which showed a small negative correlation with both the BVAQ-Cognitive and the BVAQ-Affective.

### Machine learning

Table 4 reports the MAE of the ML models for the three target alexithymia scales and the four datasets: i) the Italian training dataset (used to calibrate the ML models) and the test datasets (used only to evaluate the calibrated models); ii) the Italian test dataset; iii) the U.S. dataset; and iv) the Singaporean dataset. To offer a reference for the evaluation of the performances, we also report the MAE with 90% confidence intervals for a baseline “chance” model that always estimates the mean value of the target. Note that lower MAE values indicate a better performance on estimating the alexithymia score.

**Table 4:** Mean absolute error (MAE) with [90% confidence intervals] of the three ML models estimating the alexithymia scores (TAS-20; BVAQ-Cognitive; BVAQ-Affective) on the training subset (Italian Train Dataset) and on the three test subsets: The Italian Test Dataset, the U.S. Test Dataset and the Singaporean Test Dataset.

Target Variable	Italian Train Dataset	Italian Test Dataset	U.S. Test Dataset	Singaporean Test Dataset
**TAS-20** 0.82 [0.70–0.94]	**0.64 [0.53–0.74]**	**0.63 [0.48–0.79]**	**0.65 [0.56–0.76]**	**0.59 [0.51–0.69]**
**BVAQ-Cognitive** 0.84 [0.72–0.95]	**0.61 [0.50–0.72]**	**0.56 [0.41–0.74]**	**0.66 [0.57–0.77]**	**0.60 [0.50–0.71]**
**BVAQ-Affective** 0.82 [0.70–0.94]	**0.77 [0.66–0.90]**	**0.76 [0.55–1.02]**	**1.05 [0.90–1.21]**	**0.90 [0.75–1.06]**

In the first column, the value of MAE [with 90% confidence intervals] is reported for a baseline “chance” model that always estimates the mean value of each target variable.

BVAQ-Cognitive was the target variable best estimated (lowest MAE) within the training dataset and all the test datasets. Contrarily, BVAQ-Affective was the target variable that was most poorly estimated and did not differ substantially from the performance of the baseline “chance” model. Regarding the TAS-20, the MAE was lower than the baseline in the train set and in all the test datasets, but there was partial overlap with the baseline model.

It is worth noticing that there were no remarkable differences in the performances between the Italian train and test datasets, indicating that the calibration process was performed without biases or overfitting. Furthermore, the performance on the three test datasets was comparable on the TAS-20 and BVAQ-Cognitive, suggesting that our results were independent of the socio-cultural differences between the three samples.

By contrast, BVAQ-Affective has a performance close to baseline in the train dataset and in the Italian test dataset, and falls below the baseline in the U.S. and Singapore datasets. This result suggests a poor association between the BVAQ-Affective score and interoceptive measures.

**Fig 1:**
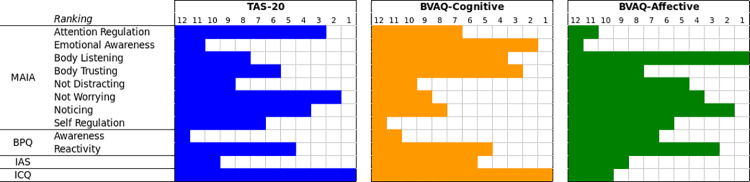
Ranking of ML predictors for each target variable (TAS-20, BVAQ-Cognitive, BVAQ-Affective), derived from the coefficients of the linear SVM model. The ranking ranges from 1 (best predictor) to 12 (worst predictor).

Fig 1 shows the ranking of predictors for each target variable. ICQ was consistently the best predictor for both the TAS-20 and BVAQ-Cognitive, the two target variables estimated above baseline levels, confirming results from the correlation analyses. By contrast, the BPQ-Awareness subscale came last for both the BVAQ-Cognitive and TAS-20, again confirming the results from the correlation analyses, which in the Italian dataset yielded no significant correlation between BPQ-Awareness and TAS-20 and found a small and barely significant correlation between BPQ-Awareness and BVAQ-Cognitive. The BPQ-Reactivity subscale was always within the first five positions, suggesting it is more important than the BPQ-Awareness subscale. As for the MAIA subscales, there was substantial variability in the rankings for different target variable estimations. For instance, MAIA Emotional Awareness came second for estimating the BVAQ-Cognitive, but second-last for the TAS-20. However, these results confirmed those obtained from the correlation analyses in the Italian sample (see S1 Table in S1 File). Finally, it is worth noticing that although IAS was significantly correlated with both the TAS-20 (r_s Italy_ = - 0.31***) and BVAQ-Cognitive (r_s Italy_ = - 0.34***), its importance in ML models was far lower than that of ICQ, which is considered to highlight the same construct, Subjective IAcc.

## Discussion

The main goal of this study was to understand if and to what extent the ability to recognize internal affective states depends on the ability to recognize internal bodily states. Therefore, we explored if, in the general population, a deficit in interoceptive ability co-occurs with higher levels of alexithymia. Our attempt to clarify the relationship between alexithymia and interoception went in two directions. Firstly, we examined whether self-reported deficits in interoception were associated with both the dimensional and categorical conceptualizations of alexithymia, respectively operationalized by the TAS-20 and the BVAQ. More specifically, we considered whether self-reported interoception levels can only estimate scores on the TAS-20 and BVAQ-Cognitive, or whether they are also an accurate estimator of BVAQ-Affective. Secondly, we aimed to clarify which components of subjective interoception are mostly closely related to alexithymia. This last objective aimed to clarify the mixed results in the literature regarding the association between subjective interoception and alexithymia, and the possibility that they derived from the different self-report instruments used to measure interoceptive ability.

Regarding the first point, our results showed that interoceptive deficits are associated with the alexithymic characteristics captured by both the TAS-20 and BVAQ-Cognitive, but not with those operationalized by the BVAQ-Affective. From our analysis of the different components that make up alexithymia construct (see S1-S3 Tables in S1 File), we can further confirm that a deficit in interoception is especially associated with both difficulty in identifying feelings and difficulty in describing feelings, which are tested in both the TAS-20 and BVAQ-Cognitive. We found a significant if smaller correlation between interoceptive deficits and those aspects of alexithymia that are considered to be more cognitive: mainly EOT (the subscale of the TAS-20 that measures a concrete and realistic style of thinking), and BVAQ Analysing (the similar, although not completely superimposable subscale of BVAQ that assesses people’s ability to explain emotional reactions). However, our findings are not completely conclusive, and they vary in in terms of specific interoceptive measures and specific samples. In particular, we found variability in the TAS-20 EOT, which had a medium association with interoceptive measures in the U.S. sample, a small association in the Italian sample, and almost no association in the Singaporean sample. Moreover, it should be noticed that, across the three samples, TAS-20 EOT didn’t reach an acceptable level of internal validity. This result is not surprising since the lower internal consistency of EOT with respect to the other two facets of TAS-20 has been widely attested, especially when administered in different linguistic and cultural contexts [48, 49]. Overall, the weaknesses highlighted for TAS-20 EOT could explain why, in the ML analysis, interoceptive measures estimated more accurately the whole BVAQ-Cognitive dimension than the TAS-20, although these measures of alexithymia are considered to be highly interchangeable.

However, our most noteworthy result was the lack of relationship between BVAQ-Affective and interoceptive measures, which was confirmed in all samples by both correlational and ML analyses. As mentioned above, fantasizing and emotionalizing factors are not included in the TAS-20 and are specific to BVAQ-Affective. This is congruent with the BVAQ authors’ idea that there are different types of alexithymia and that these are due to different dysfunctions [16]. Given these premises, it is not surprising that the interoceptive deficit was associated with only one of the two higher-order dimensions of BVAQ. What was surprising, if we adhere to the Bermond-Vorst model of alexithymia, is that the cognitive but not the affective dimension was linked to a deficit in recognizing internal bodily states. Here, we should specifically mention the Emotionalizing factor of BVAQ-Affective which was designed to capture deficits in emotional arousal and, thus, is supposed to reveal dysfunctions that occur at the lower level of physiological activation and not at the higher level of cognitive processing. Counterintuitively, besides being unrelated to interoception, both the BVAQ-Affective and its Emotionalizing subscale were found to be positively associated with the adaptive ability to not worry about physical sensations of pain and discomfort (see correlation with MAIA Not-Worrying in S1-S3 Tables in S1 File). This result raises questions about the clinical relevance of the hypothetical Affective dimension of alexithymia, which seems to fail revealing any actual dysfunction. We are specifically sceptical about a few items in the Emotionalizing subscale, which seem to capture the ability to cognitively self-regulate emotional states, thus disregarding evidence that shows alexithymia is associated with emotion dysregulation (for a recent review, see [50]).

Overall, we argue that a deficit in interoception predicts difficulty identifying and describing emotional feelings. To elaborate, this means that people who suffer from an interoceptive deficit, will also score within the upper half of the alexithymic spectrum, when levels of alexithymia are assessed by using the TAS-20 or BVAQ-Cognitive. However, different considerations apply in the case of the BVAQ-Affective. In fact, the characteristics described by this dimension do not seem to relate to any interoceptive deficit. In our opinion this result poses further challenges for the conceptualization of alexithymia proposed by the authors of the BVAQ, but does not undermine the interoceptive hypothesis of alexithymia.

Our results also allow us to say something more about which specific self-perceived interceptive deficits are related to alexithymia. Alexithymic characteristics are linked to decreased perceived capacity to detect (see IAS) and interpret (see ICQ) internal bodily signals, a deficit in the ability to direct top-down attention to regulating one’s bodily states (see MAIA), and increased symptomatology reflecting difficulties and problems of the autonomic system (see BPQ-Reactivity). The highest predictor of alexithymia levels across the three samples was the Interoceptive Confusion Questionnaire, which captures subjectively experienced difficulty in recognizing specific bodily sensations such as hunger, thirst, satiety, muscle tension, or nausea.

More specific considerations are necessary to interpret the data on the Body Perception Questionnaire (BPQ). Employing this questionnaire or misleading interpretations of its results are probably the main causes for the inconsistencies found in the literature describing the relationship between alexithymia and interoception. Indeed, while studies employing other self-reported interoceptive scales suggest that alexithymia is associated with decreased interoceptive awareness, studies using BPQ often lead to the opposite result and suggest that high levels of alexithymia are associated with hypersensitivity to bodily sensations [51], or high unspecific arousal [52] or an abnormal tendency to focus on bodily signals [53].

To account for these conflicting results, recent studies offer a more specific conceptualization of interoceptive awareness [31, 32]. They suggest that BPQ is a measure of self-perceived interoceptive attention and that this must be distinguished from self-perceived interoceptive accuracy. Following this model, while alexithymia is linked to deficits in interoceptive accuracy, it is not related to self-perceived interoceptive attention. However, this conclusion seems to be premature, especially if no other measures of interoceptive attention such as the MAIA are considered.

Some clarification is offered by Trevisan and colleagues’ meta-analysis [33], which highlights that in many studies MAIA and BPQ produce opposite results: MAIA is negatively correlated while BPQ is positively correlated with alexithymia. The results of our study furtherly clarify this point. Across our three samples, the two subscales of BPQ exhibit different patterns of results. Indeed, BPQ-Reactivity is positively associated with alexithymia, while BPQ-Awareness is not a significant predictor of alexithymia, ranking last in importance in our ML models. In our view, BPQ-Reactivity is not a measure of subjective interoceptive awareness but instead indicates some kind of malfunction in the autonomic nervous system. This explains why BPQ-Reactivity is positively correlated with higher levels of alexithymia. On the contrary, the lower predictive power of BPQ-Awareness with respect to other scales can be attributed to the confusing wording in its items, which ask respondents to state their awareness of a series of bodily signals that, as already highlighted in a recent commentary [39], are mostly unpleasant or indicative of anxiety states (e.g., stomach and gut pains, facial twitches, dry mouth, urge to urinate, cough or clear their throat, etc.). In sum, it is not clear if BPQ-Awareness asks respondents to report the frequency or awareness of these bodily signals and how people who do not experience the above-mentioned sensations might answer such questions. Overall, we argue that BPQ total scores are more likely to indicate the presence of aversive physical symptoms than to evaluate interoceptive attention. Therefore, the BPQ should not be used as a measure of subjective interoception. If this is the case, there is no reason to argue that subjective Interoceptive accuracy (Subjective IAcc) is associated with alexithymia, while subjective interoceptive attention (Subjective IAtt) is not, as was suggested by Murphy and colleagues [32]. This conclusion would definitely conflict with the results from the MAIA analysis. However, also taking the arguments presented by both Murphy [32] and Trevisan [39] into consideration, we suggest that a general focus on internal bodily sensations, estimated quantitatively on the basis of how often people report paying attention to interoceptive signals, is not per se predictive of levels of emotional awareness. Indeed, this behavioural tendency can be associated with excessive preoccupation with bodily sensations (i.e., hypochondriasis) or it can derive from the presence of painful somatic sensations that drive bottom-up attentional processes. In fact, when dispositional attention to bodily signals is qualitatively defined (by assessing the reported ability to regulate emotions through attention to body sensations, active listening to body sensations, or a tendency to not ignore sensations of pain or discomfort or to establish whether the experience of bodily sensations is perceived as trustworthy, see MAIA subscales) – then, this top-down form of interoceptive attention (also defined as “adaptive” by [39]) becomes a significant indicator of levels of emotional awareness and indicates reduced levels of alexithymia. Therefore, our MAIA results, combined with those from the meta-analysis by Trevisan and colleagues [33, 39] show that both subjective interoceptive accuracy and attention (in the above-mentioned conceptualization) are negative and significant predictors of alexithymia levels as operationalized by the TAS-20 and BVAQ-Cognitive. As a consequence, this conclusion may also debunk the common view that individuals with high levels of alexithymia are unable to report interoceptive deficits. Our findings lead in the opposite direction, although this result should be confirmed in clinical populations with higher interoceptive deficits.

In conclusion, we suggest that a weaker version of the “interoceptive hypothesis” of alexithymia would be appropriate. Our results show that interoceptive levels are good predictors of alexithymia levels. This suggests that deficits in interoception can reveal higher order impairment in the recognition of affective states. However, not all aspects of the construct of alexithymia clearly reflect an interoceptive deficit. We therefore disagree with the conclusion drawn by Brewer and colleagues [18] that “alexithymia is synonymous with interoceptive impairment”. In our view, alexithymia is neither an interoceptive impairment, nor a proxy for interoceptive impairment [27]. Indeed, even relying on the widely accepted dimensional model of alexithymia proposed by the authors of the TAS-20, we question the role played by interoceptive deficits in the cognitive characteristics of alexithymia as defined by the EOT subscale. Here, it is worth mentioning that the Externally Oriented Thinking is not only the component of the alexithymia construct with the lowest psychometric reliability, but also the one least associated with pathological variables [54]. One could conclude that a deficit in interoception is the most relevant feature of alexithymia and, thus, deserves the greatest consideration as a predictor of mental and physical disorders. However, in agreement with Taylor and Bagby [17], we believe that it is premature to characterize alexithymia as a general deficit in interoception. This strong conclusion needs further support from empirical evidence, especially within clinical populations, and requires careful consideration of the role played by different components of the alexithymia construct.

## Supporting information

S1 File Supplementary material file. Detailed information on data pre-processing and ML analysis. Correlation matrices between all scales and subscales are also displayed.
(DOCX)
