# Supplementary material for: Clarifying the relationship between alexithymia and subjective interoception
Source: PLoS One. 2021 Dec 13;16(12):e0261126. doi: 10.1371/journal.pone.0261126 (PMC8668127; doi:10.1371/journal.pone.0261126)
Supplement: S1 File — Detailed information on data pre-processing and ML analysis. Correlation matrices between all scales and subscales are also displayed. (DOCX) [file pone.0261126.s001.docx]

Supplementary Material

Clarifying the relationship between alexithymia and subjective interoception

Giulia Gaggero^1*^, Andrea Bizzego^1^, Sara Dellantonio^1^, Luigi Pastore^2^, Mengyu Lim^3^, and Gianluca Esposito^1,3,4^

^1^Department of Psychology and Cognitive Sciences, University of Trento, Rovereto, Italy

^2^Department of Education, Psychology, Communication, University of Bari, Bari, Italy

^3^Psychology Program, School of Social Sciences, Nanyang Technological University, Singapore, Singapore

^4^Lee Kong Chian School of Medicine, Nanyang Technological University, Singapore, Singapore

* Corresponding author

E-mail: giulia.gaggero@unitn.it (GG)

The below displayed are further information on the sample characteristics, on data preparation and Machine Learning analyses. Supplementary Table 1,2,3 show correlation matrices for all the scales and subscales measured within each sample.

## Participants

### **Italian Sample**

All participants from the Italian sample completed the study via Qualtrics (Provo, UT) in a unique step. Online informed consent was obtained from each participant followed by scales presentation and demographic questions. A balance Latin square design was used to present scales in a balanced order across participants. Only participants who did not fail attention-testing questions (e.g. “When answering this question, select the number four”; “Please select the answer that indicates that you are slightly in disagreement”) were able to end the survey (n =338). From this initial pool, we deleted duplicated IDs (n=2) and people declaring to have paid little attention to the study questions (n =1). Prior to data analysis, we screened survey completion time setting the lowest limit by computing the number of items (215)*3 seconds + 60 seconds for reading the instructions. No subject fell under this boundary. Further 10 subjects were excluded because they were found to be univariate or multivariate outliers and they presented multiple incongruences in their responses.

### **U.S. sample**

All measures were administered through a unique Qualtrics link (two additional scales were administered within the same link but they are not part of the present study). Compilation time was estimated to be <30 minutes. Captcha and attention checks were entered in the survey to prevent bot-like activity and to test responders’ attentiveness. A payment code was automatically provided only to those participants who arrived at the end of the survey without failing attention‐testing questions. An additional 7 subjects were excluded since they fell in the lowest 2.5 % of the distribution for compilation time. We legitimate this exclusion, considering that in literature an accepted minimum cutoff score for response time is set at 2 seconds per item [1] and the excluded participants completed the survey in a time (< 676 sec.), suggesting that they spent less than 2 seconds on each item (the entire survey consisted of informed consent, requiring at least 60 seconds to be red, plus 282 items). Further 7 subjects were excluded because they were found to be univariate or multivariate outliers and they presented multiple incongruences in their responses.

### **Singaporean Sample**

This Singaporean dataset was collected as part of a larger study approved by the IRB of the Nanyang Technological University of Singapore (NTU). All the study measures were administered to participants through three separate Qualtrics links, which could be completed at different times. The three links were presented in randomized order across participants. Only participants who completed all the three links were considered for the analysis and their data were merged by the IDs provided by researchers. Data quality was assured by screening answers given to the attention checks. A maximum of one failed attention check per link was considered as acceptable, while data from people failing more than one attention check were excluded (n = 8). Further 8 subjects were excluded because they were found to be univariate or multivariate outliers and they presented multiple incongruences in their responses.

## Data preparation

### **Missing data analysis and imputation**

Data on all items in the Italian dataset were completed. In the U.S. dataset, 3 subjects presented respectively one missing value in the BPQ scale, due to technical problems during the compilation. In the Singaporean sample, the first 43 % (n =107) of subjects missed data in two items of the BPQ Awareness subscale (<8 % of the whole BPQ Awareness subscale) and in one item of the BPQ Reactivity subscale (= 5 % of the whole BPQ Reactivity subscale), because of a researcher’s mistake in uploading the questionnaires items on the Qualtrics platform. Moreover, due to technical problems during the compilation, another subject of the Singaporean sample missed information about a further item of the BPQ Awareness subscale. In both U.S. and Singaporean sample, we considered this proportion of missing values as negligible and decided to impute our data by applying the person’s individual median in the subscale each item belonged to^[[1]](#footnote-1)^. Additionally, 2 subjects in the Singaporean group missed information about age. Descriptive analyses ignore this missing information.

### **Total Scores calculation**

Subjects’ total scores for each scale and subscale were computed following instructions given by the original validation papers and relying on the factorial structure found in the original validation studies of each measure. The data distribution of each continuous variable was checked graphically (by density plots and box-plots) and by the appropriate metrics (skewness, kurtosis, Shapiro normality test). Since distributions obtained from the sum of row scores in BPQ subscales were highly skewed, we used standardized T scores. T scores reflect a standardized value according to a normal distribution based on a mean of 50 and a standard deviation of 10. This transformation is recommended in the BPQ manual available at <https://www.stephenporges.com/body-scales> and was performed using the R code made available by authors.

### **Outlier detection and exclusion**

Both univariate and multivariate outliers were detected separately for each sample. using the *Routliers* package [2]. We detected outliers for all the independent and dependent variables entered in the Machine Learning model: TAS, BVAQ-Cognitive, BVAQ-Affective, IAS, ICQ, BPQ-Awareneess, BPQ- Reactivity and the 8 MAIA-subscales.

To screen univariate outliers, we compute median absolute deviation (MAD) for the distribution of each of the aforementioned variables in either the Italian, U.S. and Singaporean Sample, as recommended by [3]. A very conservative threshold of 3 MAD was selected to indicate univariate outliers.

To screen multivariate outliers, we used Minimum Covariance Determinant (MCD). MCD was performed separately for each sample setting alpha at 0.001. Leys et al. [4] showed MCD superiority over basic Mahalanobis distance for detecting multivariate outliers.

Cases in which univariate or multivariate outliers have been found were complexly screened. The aim of this screening was to detect uniform pattern of responding, multiple logical incongruences or other information suggesting that these cases should be excluded from analysis. If the presence of these problems was observed in more than one measure, the case was removed from the dataset.

In total, 45 univariate or multivariate outliers were found for the Italian dataset, 41 for the U.S. dataset and 30 for the Singaporean dataset. After screening for outliers, 10 subjects were eliminated from the Italian dataset, 7 were eliminated from the U.S. dataset and 8 were eliminated from the Singaporean dataset. Sample size and characteristics of the final datasets are displayed in the main manuscript.

## Descriptive and correlation analyses

Descriptive statistics of the socio-demographic and psychological data were performed separately for the Italian, American U.S. and Singaporean datasets since the main aim of this study is not to compare the samples with each other but to verify whether our results are consistent across samples presenting different socio-demographic profiles. However, for information purposes, we performed comparative analyses of variance to check for differences between samples’ means in sociodemographic and psychological measures. For each sample, assumption of normality of each continuous variable has been checked graphically and using the Shapiro-Wilk test. Since the distribution of our data did not respect univariate normality in all variables and/or datasets and because sample size differed between the three datasets, we adopted permutation tests. Here, we used a one-way permutation Analysis of Variance (ANOVA) with the *aovperm* function from the Permuco package v. 1.0.2 in R [5], setting the number of permutations at 10,000 and the alpha level at 0.05. Effect size estimates were also computed using eta squared. *Post hoc* testing used permutational independent samples *t*-tests with the *perm.t.test* function from the “RVAideMemoire” package in R [6]. To account for Type I errors introduced by multiple pairwise tests, we applied Bonferroni correction. Pearson chi-square test was also performed to compare gender between samples.

Secondly, Zero-order Spearman’s correlations between all measures were performed separately for each dataset. The p-values were corrected for multiple inference using Holm's method.

## Machine Learning Analysis

Analyses were conducted in Python using the scikit-learn library (v0.24.2). Support Vector Machines with linear kernel were used as ML models and calibrated to estimate three target variables: TAS-20 total scores, BVAQ-Cognitive and BVAQ-Affective. The variables (n = 12) used as predictors were derived from scales and subscales measuring self-reported interoceptive ability: ICQ total scores, IAS total scores, the 8 subscales from MAIA-2 (1) noticing, (2) not-distracting, (3) not-worrying, (4) attention regulation, (5) emotional regulation, (6) self-regulation, (7) body listening, (8) body trusting) and the two subscales from BPQ (BPQ-Autonomic Reactivity, BPQ-Body Awareness).

The same protocol was used to calibrate the three ML models, each one estimating one of the three target variables.

Firstly, the Italian dataset was randomly split into ‘train’ (75% of the subjects) and ‘test’ partitions (25% of the subjects). The ‘train’ partition was used to calibrate the models, while the ‘test’ partition was used to evaluate the performance of the calibrated models. The calibration of the models included the optimization of the regularization parameter C and of the weights of the variables.

The optimization was based on a traditional 10x5-fold Cross Validation scheme [7]. For each value of the regularization parameter C (C: {0.0001, 0.001, 0.01, 0.1, 1, 10, 100}) the train partition was randomly split into 5 folds: all folds except one were used to train the model which was then evaluated on the left-out fold. The procedure was iterated on the 5 folds, then repeated 10 times. The performance of the model with for each C was estimated by bootstrapping the distribution of the performance scores on the left-out fold at each iteration. The Mean Average Error (MAV) was used as performance metric. The value of C with the best performance was then selected as the optimal value. The final model was trained using the whole train dataset, using the optimal value C.

At each iteration of the optimization process, we also extracted the rankings of the predictors. At the end of the optimization process we selected the rankings corresponding to the optimal value C and computed the borda list [8] to obtain the overall importance of the predictors.

The trained model was tested on the remaining subsets: the Italian Test partition, the U.S. partition and the Singaporean partition. The performance of the model on each subset was assessed using the bootstrap to obtain the average (with 90% Confidence intervals) MAE.

# References

1. Huang JL, Curran PG, Keeney J, Poposki EM, DeShon RP Detecting and deterring insufficient effort responding to surveys. J Bus Psychol. 2012;27(1): 99-114. [doi: 10.1007/S10869-011-9231-8](doi:%2010.1007/S10869-011-9231-8)
2. Delacre M, Klein O. Routliers: Robust Outliers Detection; 2019.
3. Leys C, Ley C, Klein O, Bernard P, Licata L. Detecting Outliers: Do Not Use Standard Deviation around the Mean, Use Absolute Deviation around the Median. J Exp Soc Psychol. 2013;49: 764–766. [doi: 10.1016/j.jesp.2013.03.013](doi:%2010.1016/j.jesp.2013.03.013).
4. Leys C, Klein O, Dominicy Y, Ley C. Detecting multivariate outliers: Use a robust variant of the Mahalanobis distance. J Exp Soc Psychol. 2018;74: 150-156. [doi: 10.1016/j.jesp.2013.03.013](doi:%2010.1016/j.jesp.2013.03.013)
5. Frossard J, Renaud O. Permuco: Permutation Tests for Regression, (Repeated Measures) ANOVA/ANCOVA and Comparison of Signals. R Package Version 1.0.0; 2018.
6. Hervé MM, Hervé MM. Package ‘RVAideMemoire.’; 2020.
7. Bizzego, A., Gabrieli, G., Bornstein, M. H., Deater-Deckard, K., Lansford, J. E., Bradley, R. H., et al. Predictors of contemporary under-5 child mortality in low-and middle-income countries: a machine learning approach. Int J Environ Res. 2021;18(3): 1315.

Jurman G, Merler S, Barla A, Paoli S, Galea A, Furlanello C. Algebraic stability indicators for ranked lists in molecular profiling. Bioinformatics. 2008;24(2): 258–264. doi: 10.1093/bioinformatics/btm550

**S1 Table. Correlation matrix between all scales and subscales administered to the Italian sample (*N* = 325).** * p < .05, ** p < .01, *** p < .001.

|  | 1 | 2 | 3 | 4 | 5 | 6 | 7 | 8 | 9 | 10 | 11 | 12 | 13 | 14 | 15 | 16 | 17 | 18 | 19 | 20 | 21 | 22 | 23 | 24 | 25 | 26 | 27 |
| --- | --- | --- | --- | --- | --- | --- | --- | --- | --- | --- | --- | --- | --- | --- | --- | --- | --- | --- | --- | --- | --- | --- | --- | --- | --- | --- | --- |
| TAS |  |  |  |  |  |  |  |  |  |  |  |  |  |  |  |  |  |  |  |  |  |  |  |  |  |  |  |
| TAS_DIF | .83*** |  |  |  |  |  |  |  |  |  |  |  |  |  |  |  |  |  |  |  |  |  |  |  |  |  |  |
| TAS_DDF | .83*** | .52*** |  |  |  |  |  |  |  |  |  |  |  |  |  |  |  |  |  |  |  |  |  |  |  |  |  |
| TAS_EOT | .65*** | .31*** | .42*** |  |  |  |  |  |  |  |  |  |  |  |  |  |  |  |  |  |  |  |  |  |  |  |  |
| BVAQ | .73*** | .50*** | .70*** | .53*** |  |  |  |  |  |  |  |  |  |  |  |  |  |  |  |  |  |  |  |  |  |  |  |
| Identifying | .67*** | .77*** | .43*** | .31*** | .61*** |  |  |  |  |  |  |  |  |  |  |  |  |  |  |  |  |  |  |  |  |  |  |
| Verbalizing | .73*** | .45*** | .87*** | .42*** | .78*** | .41*** |  |  |  |  |  |  |  |  |  |  |  |  |  |  |  |  |  |  |  |  |  |
| Analyzing | .54*** | .34*** | .40*** | .59*** | .70*** | .44*** | .44*** |  |  |  |  |  |  |  |  |  |  |  |  |  |  |  |  |  |  |  |  |
| Fantasizing | .05 | -.01 | .03 | .12* | .43*** | .03 | .07 | .15** |  |  |  |  |  |  |  |  |  |  |  |  |  |  |  |  |  |  |  |
| Emotionalizing | .02 | -.20*** | .13* | .20*** | .35*** | -.17** | .17** | .17** | .09 |  |  |  |  |  |  |  |  |  |  |  |  |  |  |  |  |  |  |
| BVAQ_Cognitive | .84*** | .67*** | .77*** | .53*** | .89*** | .77*** | .83*** | .72*** | .10 | .07 |  |  |  |  |  |  |  |  |  |  |  |  |  |  |  |  |  |
| BVAQ_Affective | .07 | -.10 | .12* | .21*** | .55*** | -.07 | .18*** | .23*** | .79*** | .65*** | .14* |  |  |  |  |  |  |  |  |  |  |  |  |  |  |  |  |
| IAS | -.31*** | -.33*** | -.25*** | -.10 | -.28*** | -.40*** | -.21*** | -.24*** | .02 | .01 | -.34*** | -.00 |  |  |  |  |  |  |  |  |  |  |  |  |  |  |  |
| ICQ | .46*** | .50*** | .36*** | .18*** | .39*** | .53*** | .33*** | .34*** | -.10 | -.02 | .50*** | -.06 | -.53*** |  |  |  |  |  |  |  |  |  |  |  |  |  |  |
| BPQ | .04 | .14** | .02 | -.14* | -.10 | .08 | -.04 | -.17** | -.16** | -.15** | -.03 | -.20*** | .10 | .10 |  |  |  |  |  |  |  |  |  |  |  |  |  |
| BPQ_A | -.09 | -.03 | -.05 | -.16** | -.21*** | -.10 | -.12* | -.25*** | -.15** | -.10 | -.17** | -.17** | .26*** | -.08 | .91*** |  |  |  |  |  |  |  |  |  |  |  |  |
| BPQ_R | .28*** | .45*** | .14** | -.02 | .15** | .45*** | .13* | .05 | -.10 | -.18*** | .26*** | -.17** | -.31*** | .39*** | .62*** | .29*** |  |  |  |  |  |  |  |  |  |  |  |
| BPQ_R_Supra | .25*** | .40*** | .14** | -.03 | .15** | .41*** | .14* | .06 | -.10 | -.14* | .25*** | -.14* | -.29*** | .37*** | .56*** | .23*** | .94*** |  |  |  |  |  |  |  |  |  |  |
| BPQ_R_Sub | .23*** | .37*** | .09 | -.01 | .08 | .35*** | .06 | .01 | -.08 | -.22*** | .19*** | -.19*** | -.24*** | .29*** | .55*** | .31*** | .77*** | .53*** |  |  |  |  |  |  |  |  |  |
| MAIA_tot | -.40*** | -.37*** | -.27*** | -.29*** | -.35*** | -.45*** | -.28*** | -.33*** | -.03 | .13* | -.44*** | .03 | .35*** | -.36*** | .19*** | .23*** | -.03 | -.02 | -.02 |  |  |  |  |  |  |  |  |
| MAIA_AttentionReg | -.29*** | -.28*** | -.16** | -.25*** | -.26*** | -.36*** | -.17** | -.27*** | -.06 | .15** | -.34*** | .03 | .27*** | -.29*** | .12* | .17** | -.06 | -.03 | -.07 | .68*** |  |  |  |  |  |  |  |
| MAIA_BodyListening | -.30*** | -.21*** | -.24*** | -.27*** | -.35*** | -.32*** | -.27*** | -.35*** | -.09 | .04 | -.39*** | -.06 | .29*** | -.30*** | .23*** | .24*** | .08 | .07 | .08 | .80*** | .50*** |  |  |  |  |  |  |
| MAIA_BodyTrusting | -.35*** | -.40*** | -.25*** | -.15** | -.29*** | -.43*** | -.26*** | -.23*** | .03 | .13* | -.39*** | .09 | .26*** | -.33*** | .05 | .13* | -.18*** | -.19*** | -.12* | .73*** | .42*** | .53*** |  |  |  |  |  |
| MAIA_EmoAwareness | -.18** | -.07 | -.14* | -.26*** | -.24*** | -.18** | -.16** | -.27*** | -.05 | -.10 | -.24*** | -.11* | .29*** | -.19*** | .29*** | .24*** | .21*** | .22*** | .16** | .64*** | .38*** | .57*** | .27*** |  |  |  |  |
| MAIA_NotDistracting | -.15** | -.16** | -.14* | -.05 | -.19*** | -.16** | -.22*** | -.09 | -.05 | -.01 | -.21*** | -.07 | .08 | -.25*** | -.02 | -.00 | -.04 | -.01 | -.10 | .18** | .07 | .08 | .07 | -.01 |  |  |  |
| MAIA_Noticing | -.22*** | -.09 | -.18*** | -.27*** | -.28*** | -.19*** | -.18** | -.29*** | -.08 | -.10 | -.26*** | -.14** | .33*** | -.21*** | .33*** | .30*** | .18** | .18*** | .14* | .53*** | .35*** | .44*** | .18** | .53*** | .07 |  |  |
| MAIA_NotWorrying | -.18** | -.30*** | -.05 | -.02 | .04 | -.22*** | -.01 | .05 | .04 | .36*** | -.07 | .23*** | -.06 | .04 | -.17** | -.08 | -.27*** | -.26*** | -.21*** | .23*** | .15** | -.05 | .17** | -.16** | -.08 | -.13* |  |
| MAIA_SelfReg | -.28*** | -.28*** | -.19*** | -.20*** | -.18** | -.29*** | -.18** | -.13* | .04 | .12* | -.26*** | .08 | .22*** | -.21*** | .08 | .10 | -.03 | -.03 | -.01 | .77*** | .45*** | .55*** | .60*** | .40*** | -.02 | .24*** | .22*** |

**S2 Table. Correlation matrix between all scales and subscales administered to the U.S. sample (*N* = 250).** * p < .05, ** p < .01, *** p < .001.

|  | 1 | 2 | 3 | 4 | 5 | 6 | 7 | 8 | 9 | 10 | 11 | 12 | 13 | 14 | 15 | 16 | 17 | 18 | 19 | 20 | 21 | 22 | 23 | 24 | 25 | 26 | 27 |
| --- | --- | --- | --- | --- | --- | --- | --- | --- | --- | --- | --- | --- | --- | --- | --- | --- | --- | --- | --- | --- | --- | --- | --- | --- | --- | --- | --- |
| TAS |  |  |  |  |  |  |  |  |  |  |  |  |  |  |  |  |  |  |  |  |  |  |  |  |  |  |  |
| TAS_DIF | .88*** |  |  |  |  |  |  |  |  |  |  |  |  |  |  |  |  |  |  |  |  |  |  |  |  |  |  |
| TAS_DDF | .86*** | .66*** |  |  |  |  |  |  |  |  |  |  |  |  |  |  |  |  |  |  |  |  |  |  |  |  |  |
| TAS_EOT | .71*** | .41*** | .46*** |  |  |  |  |  |  |  |  |  |  |  |  |  |  |  |  |  |  |  |  |  |  |  |  |
| BVAQ | .74*** | .58*** | .62*** | .64*** |  |  |  |  |  |  |  |  |  |  |  |  |  |  |  |  |  |  |  |  |  |  |  |
| Identifying | .76*** | .78*** | .59*** | .47*** | .67*** |  |  |  |  |  |  |  |  |  |  |  |  |  |  |  |  |  |  |  |  |  |  |
| Verbalizing | .68*** | .47*** | .82*** | .41*** | .65*** | .48*** |  |  |  |  |  |  |  |  |  |  |  |  |  |  |  |  |  |  |  |  |  |
| Analyzing | .60*** | .46*** | .41*** | .64*** | .82*** | .54*** | .42*** |  |  |  |  |  |  |  |  |  |  |  |  |  |  |  |  |  |  |  |  |
| Fantasizing | .16** | .11 | -.01 | .36*** | .54*** | .18** | -.08 | .40*** |  |  |  |  |  |  |  |  |  |  |  |  |  |  |  |  |  |  |  |
| Emotionalizing | .04 | -.05 | -.01 | .16* | .47*** | -.08 | .08 | .38*** | .30*** |  |  |  |  |  |  |  |  |  |  |  |  |  |  |  |  |  |  |
| BVAQ_Cognitive | .85*** | .70*** | .78*** | .61*** | .87*** | .81*** | .82*** | .76*** | .16* | .15* |  |  |  |  |  |  |  |  |  |  |  |  |  |  |  |  |  |
| BVAQ_Affective | .14* | .06 | .00 | .32*** | .64*** | .08 | .01 | .48*** | .85*** | .74*** | .20** |  |  |  |  |  |  |  |  |  |  |  |  |  |  |  |  |
| IAS | -.34*** | -.33*** | -.26*** | -.22*** | -.25*** | -.38*** | -.22*** | -.20** | .03 | .04 | -.32*** | .02 |  |  |  |  |  |  |  |  |  |  |  |  |  |  |  |
| ICQ | .69*** | .71*** | .55*** | .37*** | .52*** | .65*** | .39*** | .49*** | .05 | -.00 | .62*** | .05 | -.47*** |  |  |  |  |  |  |  |  |  |  |  |  |  |  |
| BPQ | .27*** | .31*** | .14* | .21*** | .12 | .25*** | .03 | .15* | .05 | -.11 | .18** | -.04 | .01 | .30*** |  |  |  |  |  |  |  |  |  |  |  |  |  |
| BPQ_A | .07 | .10 | .00 | .09 | -.04 | .07 | -.05 | .02 | -.01 | -.12* | .02 | -.09 | .13* | .06 | .91*** |  |  |  |  |  |  |  |  |  |  |  |  |
| BPQ_R | .46*** | .55*** | .31*** | .25*** | .26*** | .44*** | .18** | .28*** | -.00 | -.12 | .37*** | -.07 | -.28*** | .59*** | .62*** | .31*** |  |  |  |  |  |  |  |  |  |  |  |
| BPQ_R_Supra | .48*** | .55*** | .33*** | .27*** | .31*** | .47*** | .21*** | .32*** | .01 | -.08 | .40*** | -.04 | -.29*** | .59*** | .58*** | .27*** | .96*** |  |  |  |  |  |  |  |  |  |  |
| BPQ_R_Sub | .32*** | .40*** | .19** | .17** | .12 | .28*** | .07 | .13* | .00 | -.16* | .20** | -.09 | -.19** | .42*** | .55*** | .31*** | .80*** | .62*** |  |  |  |  |  |  |  |  |  |
| MAIA_tot | -.42*** | -.34*** | -.42*** | -.28*** | -.30*** | -.42*** | -.45*** | -.25*** | .12 | .19** | -.46*** | .18** | .40*** | -.34*** | .13* | .20** | -.15* | -.13* | -.13* |  |  |  |  |  |  |  |  |
| MAIA_AttentionReg | -.23*** | -.14* | -.28*** | -.17** | -.19** | -.26*** | -.35*** | -.16** | .12 | .15* | -.32*** | .15* | .33*** | -.22*** | .13* | .17** | -.08 | -.08 | -.04 | .79*** |  |  |  |  |  |  |  |
| MAIA_BodyListening | -.25*** | -.13* | -.33*** | -.19** | -.24*** | -.23*** | -.40*** | -.21*** | .10 | .04 | -.34*** | .08 | .27*** | -.13* | .28*** | .28*** | .05 | .05 | .05 | .80*** | .62*** |  |  |  |  |  |  |
| MAIA_BodyTrusting | -.39*** | -.35*** | -.35*** | -.24*** | -.24*** | -.44*** | -.36*** | -.19** | .11 | .23*** | -.42*** | .20** | .40*** | -.29*** | -.03 | .04 | -.25*** | -.22*** | -.22*** | .78*** | .57*** | .52*** |  |  |  |  |  |
| MAIA_EmoAwareness | -.20** | -.12 | -.20** | -.17** | -.27*** | -.22*** | -.22*** | -.24*** | -.04 | -.10 | -.27*** | -.10 | .32*** | -.18** | .25*** | .29*** | .02 | .02 | .05 | .66*** | .50*** | .60*** | .41*** |  |  |  |  |
| MAIA_NotDistracting | -.31*** | -.38*** | -.19** | -.17** | -.16* | -.29*** | -.17** | -.18** | .07 | .07 | -.25*** | .08 | .20** | -.35*** | -.17** | -.05 | -.33*** | -.30*** | -.29*** | .29*** | .11 | .14* | .14* | .01 |  |  |  |
| MAIA_Noticing | -.24*** | -.16** | -.21*** | -.21*** | -.28*** | -.31*** | -.23*** | -.26*** | .02 | -.05 | -.32*** | -.01 | .36*** | -.25*** | .24*** | .27*** | .02 | -.00 | .06 | .59*** | .55*** | .47*** | .39*** | .54*** | -.08 |  |  |
| MAIA_NotWorrying | -.23*** | -.27*** | -.22*** | -.04 | .04 | -.14* | -.13* | .07 | .08 | .38*** | -.10 | .28*** | -.02 | -.16** | -.21*** | -.16** | -.25*** | -.17** | -.33*** | .26*** | .14* | -.02 | .26*** | -.12 | .07 | -.08 |  |
| MAIA_SelfReg | -.34*** | -.28*** | -.37*** | -.19** | -.21*** | -.33*** | -.42*** | -.17** | .19** | .20** | -.39*** | .23*** | .32*** | -.28*** | .07 | .10 | -.14* | -.13* | -.10 | .82*** | .65*** | .61*** | .65*** | .48*** | .14* | .39*** | .22*** |

**S3 Table. Correlation matrix between all scales and subscales administered to the Singaporean sample (*N* = 239).** * p < .05, ** p < .01, *** p < .001.

|  | 1 | 2 | 3 | 4 | 5 | 6 | 7 | 8 | 9 | 10 | 11 | 12 | 13 | 14 | **15** | **16** | **17** | **18** | **19** | 20 | 21 | 22 | 23 | 24 | 25 | 26 | 27 |
| --- | --- | --- | --- | --- | --- | --- | --- | --- | --- | --- | --- | --- | --- | --- | --- | --- | --- | --- | --- | --- | --- | --- | --- | --- | --- | --- | --- |
| TAS |  |  |  |  |  |  |  |  |  |  |  |  |  |  |  |  |  |  |  |  |  |  |  |  |  |  |  |
| TAS_DIF | .84*** |  |  |  |  |  |  |  |  |  |  |  |  |  |  |  |  |  |  |  |  |  |  |  |  |  |  |
| TAS_DDF | .84*** | .61*** |  |  |  |  |  |  |  |  |  |  |  |  |  |  |  |  |  |  |  |  |  |  |  |  |  |
| TAS_EOT | .58*** | .22*** | .27*** |  |  |  |  |  |  |  |  |  |  |  |  |  |  |  |  |  |  |  |  |  |  |  |  |
| BVAQ | .48*** | .30*** | .50*** | .32*** |  |  |  |  |  |  |  |  |  |  |  |  |  |  |  |  |  |  |  |  |  |  |  |
| Identifying | .55*** | .63*** | .45*** | .10 | .47*** |  |  |  |  |  |  |  |  |  |  |  |  |  |  |  |  |  |  |  |  |  |  |
| Verbalizing | .58*** | .37*** | .76*** | .18** | .68*** | .35*** |  |  |  |  |  |  |  |  |  |  |  |  |  |  |  |  |  |  |  |  |  |
| Analyzing | .42*** | .24*** | .37*** | .39*** | .70*** | .26*** | .42*** |  |  |  |  |  |  |  |  |  |  |  |  |  |  |  |  |  |  |  |  |
| Fantasizing | .01 | -.07 | -.06 | .19** | .42*** | -.01 | -.04 | .11 |  |  |  |  |  |  |  |  |  |  |  |  |  |  |  |  |  |  |  |
| Emotionalizing | -.16* | -.30*** | -.13* | .15* | .47*** | -.18** | .09 | .32*** | .19** |  |  |  |  |  |  |  |  |  |  |  |  |  |  |  |  |  |  |
| BVAQ_Cognitive | .68*** | .53*** | .72*** | .28*** | .83*** | .67*** | .84*** | .68*** | .02 | .10 |  |  |  |  |  |  |  |  |  |  |  |  |  |  |  |  |  |
| BVAQ_Affective | -.11 | -.24*** | -.14* | .19** | .57*** | -.12 | .02 | .25*** | .78*** | .72*** | .06 |  |  |  |  |  |  |  |  |  |  |  |  |  |  |  |  |
| IAS | -.22*** | -.28*** | -.10 | -.12 | -.17** | -.44*** | -.08 | -.09 | .08 | -.05 | -.24*** | .02 |  |  |  |  |  |  |  |  |  |  |  |  |  |  |  |
| ICQ | .46*** | .50*** | .39*** | .12 | .25*** | .49*** | .33*** | .23*** | -.17* | -.08 | .46*** | -.19** | -.46*** |  |  |  |  |  |  |  |  |  |  |  |  |  |  |
| **BPQ** | **.12** | **.14*** | **.14*** | **-.03** | **-.05** | **.03** | **.08** | **-.00** | **-.14*** | **-.18**** | **.06** | **-.21**** | **.09** | **.03** |  |  |  |  |  |  |  |  |  |  |  |  |  |
| **BPQ_Awareness** | **-.02** | **-.03** | **.02** | **-.04** | **-.12** | **-.14*** | **-.01** | **-.08** | **-.10** | **-.13** | **-.09** | **-.15*** | **.20**** | **-.17**** | **.89***** |  |  |  |  |  |  |  |  |  |  |  |  |
| **BPQ_Reactivity** | **.38***** | **.43***** | **.31***** | **.08** | **.14*** | **.39***** | **.22***** | **.18**** | **-.12** | **-.19**** | **.33***** | **-.21**** | **-.21**** | **.41***** | **.55***** | **.15*** |  |  |  |  |  |  |  |  |  |  |  |
| **BPQ_R_Supra** | **.37***** | **.40***** | **.32***** | **.09** | **.17**** | **.38***** | **.24***** | **.17**** | **-.09** | **-.15*** | **.33***** | **-.16*** | **-.19**** | **.41***** | **.51***** | **.12** | **.95***** |  |  |  |  |  |  |  |  |  |  |
| **BPQ_R_Sub** | **.28***** | **.34***** | **.21**** | **.06** | **.02** | **.27***** | **.11** | **.13*** | **-.15*** | **-.21***** | **.19**** | **-.24***** | **-.19**** | **.29***** | **.49***** | **.18**** | **.83***** | **.63***** |  |  |  |  |  |  |  |  |  |
| MAIA_tot | -.36*** | -.33*** | -.28*** | -.16* | -.27*** | -.50*** | -.26*** | -.21*** | .05 | .11 | -.43*** | .11 | .38*** | -.44*** | **.09** | **.18**** | **-.16*** | **-.17**** | **-.08** |  |  |  |  |  |  |  |  |
| MAIA_AttentionReg | -.18** | -.11 | -.15* | -.10 | -.11 | -.26*** | -.13* | -.10 | -.01 | .17** | -.21** | .12 | .28*** | -.26*** | **.11** | **.14*** | **-.01** | **-.01** | **.01** | .66*** |  |  |  |  |  |  |  |
| MAIA_BodyListening | -.10 | -.06 | -.10 | -.04 | -.10 | -.22*** | -.12 | -.08 | .08 | .06 | -.20** | .08 | .15* | -.20** | **.10** | **.11** | **-.02** | **.00** | **-.03** | .68*** | .41*** |  |  |  |  |  |  |
| MAIA_BodyTrusting | -.36*** | -.37*** | -.26*** | -.18** | -.34*** | -.40*** | -.29*** | -.26*** | -.05 | -.03 | -.40*** | -.05 | .35*** | -.39*** | **.02** | **.13*** | **-.22***** | **-.24***** | **-.15*** | .65*** | .31*** | .37*** |  |  |  |  |  |
| MAIA_EmoAwareness | -.10 | -.05 | -.05 | -.13* | -.17** | -.29*** | -.03 | -.09 | -.12 | -.02 | -.17** | -.10 | .30*** | -.17** | **.18**** | **.16*** | **.05** | **.01** | **.10** | .65*** | .46*** | .48*** | .24*** |  |  |  |  |
| MAIA_NotDistracting | -.30*** | -.26*** | -.31*** | -.13* | -.15* | -.09 | -.29*** | -.14* | .19** | .01 | -.25*** | .12 | .03 | -.25*** | **-.09** | **.02** | **-.20**** | **-.20**** | **-.12** | .06 | -.13* | -.09 | .02 | -.20** |  |  |  |
| MAIA_Noticing | -.03 | -.04 | .06 | -.04 | -.12 | -.24*** | .01 | -.08 | -.10 | -.03 | -.13* | -.06 | .28*** | -.23*** | **.17**** | **.17**** | **.04** | **.02** | **.08** | .59*** | .44*** | .40*** | .24*** | .52*** | -.22*** |  |  |
| MAIA_NotWorrying | -.25*** | -.26*** | -.21** | -.10 | .08 | -.18** | -.06 | -.00 | .08 | .37*** | -.09 | .29*** | .04 | -.13* | **-.14*** | **-.03** | **-.22***** | **-.19**** | **-.21***** | .27*** | .16* | -.06 | .12 | -.04 | .06 | -.02 |  |
| MAIA_SelfReg | -.25*** | -.29*** | -.18** | -.05 | -.18** | -.45*** | -.16* | -.13* | .10 | .10 | -.32*** | .15* | .30*** | -.27*** | **.01** | **.06** | **-.10** | **-.11** | **-.09** | .75*** | .50*** | .44*** | .46*** | .46*** | -.13* | .42*** | .15* |

**S4 Table. Correlation matrix between all scales and subscales administered to the Singaporean sample (*N*= 238) without imputation of missing values.** Value at BPQ scale and subscales were computed without considering the three items of BPQ containing the 43 % of missing values. One subject was also excluded from this analysis because he/she contained a missing value in a further BPQ item. * p < .05, ** p < .01, *** p < .001.

|  | 1 | 2 | 3 | 4 | 5 | 6 | 7 | 8 | 9 | 10 | 11 | 12 | 13 | 14 | 15 | 16 | 17 | 18 | 19 | 20 | 21 | 22 | 23 | 24 | 25 | 26 | 27 |
| --- | --- | --- | --- | --- | --- | --- | --- | --- | --- | --- | --- | --- | --- | --- | --- | --- | --- | --- | --- | --- | --- | --- | --- | --- | --- | --- | --- |
| TAS |  |  |  |  |  |  |  |  |  |  |  |  |  |  |  |  |  |  |  |  |  |  |  |  |  |  |  |
| TAS_DIF | .84*** |  |  |  |  |  |  |  |  |  |  |  |  |  |  |  |  |  |  |  |  |  |  |  |  |  |  |
| TAS_DDF | .84*** | .62*** |  |  |  |  |  |  |  |  |  |  |  |  |  |  |  |  |  |  |  |  |  |  |  |  |  |
| TAS_EOT | .59*** | .25* | .29*** |  |  |  |  |  |  |  |  |  |  |  |  |  |  |  |  |  |  |  |  |  |  |  |  |
| BVAQ | .47*** | .30*** | .48*** | .31*** |  |  |  |  |  |  |  |  |  |  |  |  |  |  |  |  |  |  |  |  |  |  |  |
| Identifying | .56*** | .64*** | .45*** | .13 | .47*** |  |  |  |  |  |  |  |  |  |  |  |  |  |  |  |  |  |  |  |  |  |  |
| Verbalizing | .54*** | .34*** | .73*** | .16 | .69*** | .33*** |  |  |  |  |  |  |  |  |  |  |  |  |  |  |  |  |  |  |  |  |  |
| Analyzing | .40*** | .24* | .36*** | .38*** | .71*** | .27** | .43*** |  |  |  |  |  |  |  |  |  |  |  |  |  |  |  |  |  |  |  |  |
| Fantasizing | .01 | -.06 | -.06 | .18 | .43*** | -.00 | -.03 | .13 |  |  |  |  |  |  |  |  |  |  |  |  |  |  |  |  |  |  |  |
| Emotionalizing | -.17 | -.30*** | -.14 | .12 | .46*** | -.18 | .09 | .33*** | .18 |  |  |  |  |  |  |  |  |  |  |  |  |  |  |  |  |  |  |
| BVAQ_Cognitive | .66*** | .52*** | .70*** | .27** | .83*** | .68*** | .84*** | .69*** | .03 | .11 |  |  |  |  |  |  |  |  |  |  |  |  |  |  |  |  |  |
| BVAQ_Affective | -.11 | -.23 | -.15 | .17 | .57*** | -.12 | .02 | .27** | .79*** | .71*** | .06 |  |  |  |  |  |  |  |  |  |  |  |  |  |  |  |  |
| IAS | -.23 | -.28** | -.12 | -.13 | -.15 | -.43*** | -.08 | -.07 | .10 | -.02 | -.23 | .05 |  |  |  |  |  |  |  |  |  |  |  |  |  |  |  |
| ICQ | .47*** | .50*** | .40*** | .13 | .25* | .50*** | .32*** | .23* | -.16 | -.08 | .46*** | -.19 | -.46*** |  |  |  |  |  |  |  |  |  |  |  |  |  |  |
| **BPQ** | **.13** | **.15** | **.16** | **-.03** | **-.01** | **.04** | **.12** | **.02** | **-.12** | **-.15** | **.09** | **-.19** | **.07** | **.06** |  |  |  |  |  |  |  |  |  |  |  |  |  |
| **BPQ_Awareness** | **-.01** | **-.02** | **.04** | **-.05** | **-.10** | **-.14** | **.02** | **-.07** | **-.09** | **-.10** | **-.07** | **-.13** | **.20** | **-.15** | **.88***** |  |  |  |  |  |  |  |  |  |  |  |  |
| **BPQ_Reactivity** | **.37***** | **.41***** | **.31***** | **.09** | **.13** | **.38***** | **.23** | **.16** | **-.15** | **-.19** | **.32***** | **-.23** | **-.24*** | **.41***** | **.55***** | **.15** |  |  |  |  |  |  |  |  |  |  |  |
| **BPQ_R_Supra** | **.36***** | **.37***** | **.32***** | **.10** | **.16** | **.37***** | **.24*** | **.16** | **-.13** | **-.15** | **.33***** | **-.19** | **-.22** | **.40***** | **.51***** | **.12** | **.96***** |  |  |  |  |  |  |  |  |  |  |
| **BPQ_R_Sub** | **.28**** | **.33***** | **.22** | **.06** | **.01** | **.27**** | **.12** | **.12** | **-.17** | **-.22** | **.19** | **-.26**** | **-.23*** | **.30***** | **.49***** | **.19** | **.82***** | **.65***** |  |  |  |  |  |  |  |  |  |
| MAIA_tot | -.37*** | -.35*** | -.28** | -.17 | -.26* | -.51*** | -.24* | -.20 | .06 | .14 | -.41*** | .13 | .40*** | -.44*** | **.08** | **.17** | **-.18** | **-.18** | **-.12** |  |  |  |  |  |  |  |  |
| MAIA_AttentionReg | -.21 | -.14 | -.16 | -.12 | -.10 | -.27** | -.12 | -.09 | .01 | .18 | -.21 | .14 | .30*** | -.27** | **.10** | **.13** | **-.05** | **-.04** | **-.04** | .69*** |  |  |  |  |  |  |  |
| MAIA_BodyListening | -.12 | -.09 | -.12 | -.06 | -.10 | -.24* | -.11 | -.08 | .09 | .08 | -.20 | .09 | .18 | -.21 | **.11** | **.12** | **-.06** | **-.04** | **-.07** | .69*** | .43*** |  |  |  |  |  |  |
| MAIA_BodyTrusting | -.36*** | -.37*** | -.26** | -.18 | -.33*** | -.41*** | -.29** | -.26* | -.05 | -.01 | -.41*** | -.04 | .36*** | -.40*** | **.01** | **.13** | **-.24*** | **-.25*** | **-.17** | .66*** | .34*** | .39*** |  |  |  |  |  |
| MAIA_EmoAwareness | -.11 | -.06 | -.05 | -.15 | -.17 | -.29*** | -.04 | -.08 | -.11 | .00 | -.17 | -.08 | .29*** | -.15 | **.17** | **.17** | **.03** | **-.01** | **.09** | .65*** | .47*** | .48*** | .24* |  |  |  |  |
| MAIA_NotDistracting | -.29*** | -.26* | -.30*** | -.12 | -.18 | -.10 | -.31*** | -.17 | .15 | -.01 | -.26** | .08 | .01 | -.26* | **-.15** | **-.05** | **-.19** | **-.18** | **-.11** | .05 | -.13 | -.09 | .02 | -.20 |  |  |  |
| MAIA_Noticing | -.06 | -.07 | .04 | -.07 | -.12 | -.26* | .03 | -.07 | -.10 | -.01 | -.13 | -.05 | .27** | -.22 | **.18** | **.19** | **.05** | **.03** | **.09** | .57*** | .43*** | .39*** | .22 | .52*** | -.23 |  |  |
| MAIA_NotWorrying | -.23 | -.24* | -.19 | -.08 | .08 | -.16 | -.07 | .00 | .07 | .35*** | -.08 | .26** | .04 | -.11 | **-.14** | **-.04** | **-.21** | **-.17** | **-.21** | .27** | .17 | -.05 | .13 | -.03 | .07 | -.04 |  |
| MAIA_SelfReg | -.27** | -.31*** | -.20 | -.07 | -.17 | -.46*** | -.15 | -.12 | .11 | .13 | -.31*** | .17 | .32*** | -.28** | **.01** | **.07** | **-.13** | **-.13** | **-.12** | .77*** | .53*** | .46*** | .49*** | .47*** | -.12 | .41*** | .16 |

1. For the Singaporean sample we also computed total scores at BPQ, BPQ A and BPQ R by omitting the three items that were missed by the 43 % of the sample. Correlation coefficients between BPQ and BPQ subscales and the other scales are reported below in Supplementary Table 4. Supplementary Table 4 and Supplementary Table 3 report comparable correlation coefficients, suggesting that the imputation of missing values did not impact the results of correlation analysis. [↑](#footnote-ref-1)
